# Supplementary figures and images for: Social Isolation Does Not Alter Exploratory Behaviour, Spatial Learning and Memory in Captive Damaraland Mole-Rats (Fukomys damarensis)
Source: Animals (Basel). 2023 Feb 3;13(3):543. doi: 10.3390/ani13030543 (PMC9913580; doi:10.3390/ani13030543)

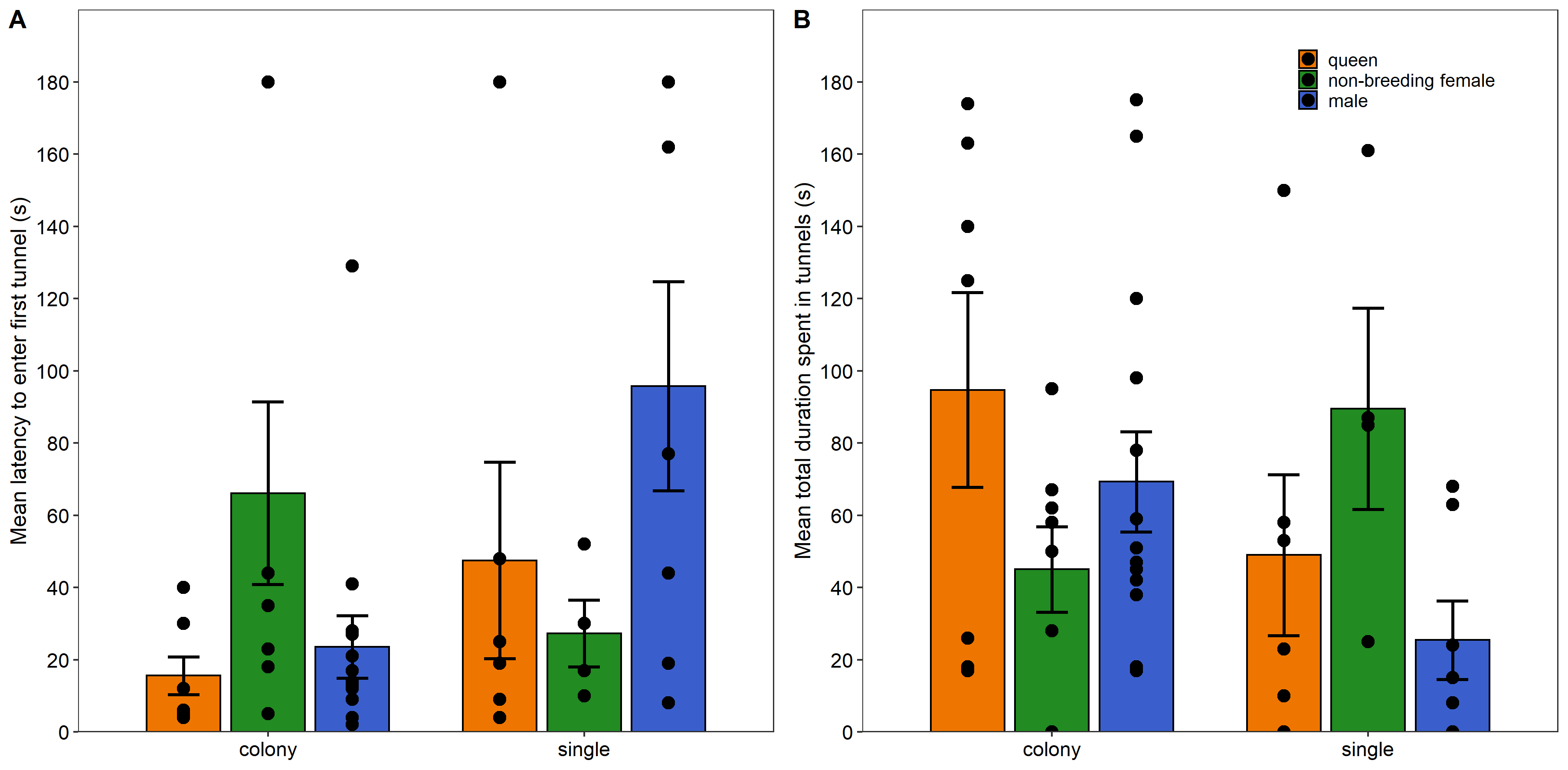

Supplement: Supplementary file 1 [file animals-13-00543-s001.zip › Supp Fig1.tiff]

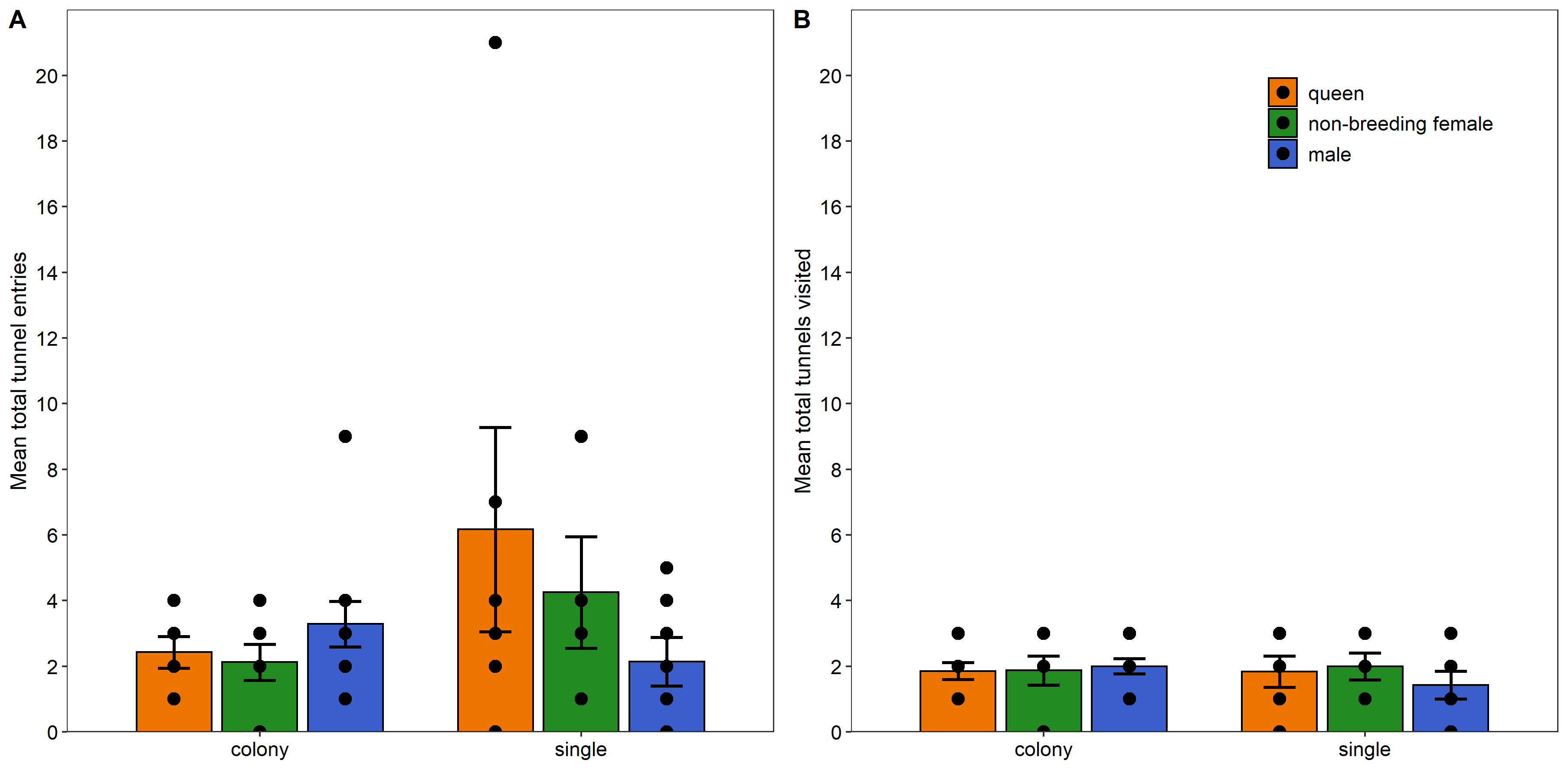

Supplement: Supplementary file 1 [file animals-13-00543-s001.zip › Supp Fig2.tiff]
